# Overexpression of *ThVHAc1* and its potential upstream regulator, *ThWRKY7*, improved plant tolerance of Cadmium stress

**DOI:** 10.1038/srep18752

**Published:** 2016-01-08

**Authors:** Guiyan Yang, Chao Wang, Yucheng Wang, Yucong Guo, Yulin Zhao, Chuanping Yang, Caiqiu Gao

**Affiliations:** 1 State Key Laboratory of Tree Genetics and Breeding (Northeast Forestry University), 26 Hexing Road, Harbin 150040, China; 2 Laboratory of Walnut Research Center, College of Forestry, Northwest A & F University, Yangling, 712100 Shaanxi, China

## Abstract

As one of the most toxic heavy metals in the environment, cadmium (Cd) poses a severe threat to plant growth. We previously reported that overexpression of the *Tamarix hispida* V-ATPase c subunit (*ThVHAc1*) improved the Cd tolerance of *Saccharomyces cerevisiae*. In the current study, we further explored the Cd tolerance conferred by *ThVHAc1* in *Arabidopsis* and *T. hispida*. *ThVHAc1* transgenic *Arabidopsis* had higher seed germination, biomass, and chlorophyll content under CdCl_2_ treatment. In Cd-stressed plants, overexpression of *ThVHAc1* significantly improved V-ATPase activity and affected the expression of other V-ATPase subunit-encoding genes. Intriguingly, the lower level of ROS accumulation in *ThVHAc1-*overexpressing lines under CdCl_2_ treatment demonstrated that *ThVHAc1* may modulate Cd stress tolerance by regulating ROS homeostasis. Transient expression of *ThVHAc1* in *T. hispida* further confirmed these findings. Furthermore, promoter analysis and yeast one-hybrid assay revealed that the transcription factor *ThWRKY7* can specifically bind to the WRKY *cis*-element in the *ThVHAc1* promoter*. ThWRKY7* exhibited similar expression patterns as *ThVHAc1* under CdCl_2_ treatment and improved Cd tolerance, suggesting that *ThWRKY7* may be an upstream regulatory gene of *ThVHAc1.* Therefore, our results show that the combination of *ThVHAc1* and its upstream regulator could be used to improve Cd stress tolerance in woody plants.

Unlike other heavy metals that function as micronutrients (such as Fe, Mn and Mo) or trace elements (such as Cu, Zn, Ni and W), Cd, Pb, Hg, Ag and U have no known nutritional function and are toxic to plants^1^. These toxicities can cause many detrimental effects, including modification of protein profiles, reduced plant growth, decreased seed germination rates, the induction of reactive oxygen species (ROS) production, cell death, chlorosis/necrosis, and turgor loss^1^,^2^,^3^. Furthermore, the high solubility of heavy metals in water results in uptake by plants, which can cause serious harm to the food chain and human health.

Cd is regarded as one of the most phytotoxic heavy metals. In a majority of soil samples, Ni, Cu, Co, Zn, Se, Pb, and Cr have been found to be moderately enriched, whereas Sb and Cd were extremely highly enriched^4^. Because soil Cd contamination affects the ceramic industry^5^, agricultural fields^6^, and the growth of sea rocket^7^, among others, understanding the molecular mechanisms of plants’ responses to Cd stress is crucial for improving plants’ adjustment and/or adaptation to Cd stress^8^,^9^,^10^. Functional genomics technologies and protein analysis methods have been used to broaden our knowledge of the pathways that respond to Cd stress in plants^8^. For example, overexpression of *BjCdR15/TGA3*, a bZIP transcription factor, effectively improved Cd uptake by roots and enhanced Cd tolerance in *Arabidopsis* and tobacco^9^. Moreover, *AtMYB4* in *Arabidopsis* and *WRKY53* in *Thlaspi caerulescens* have also been reported to play a potential role in Cd stress response^11^,^12^.

In plants, the regulation of Cd tolerance involves several enzymes, including ATPase, HMA2, and HMA4, which are essential for Cd transport^13^. Cd is known to affect the activity of plasma membrane ATPase^14^. The V-ATPase motive force-dependent cation antiporter can significantly contribute to Cd detoxification by vacuolar compartmentalization in barley roots^15^. V-ATPase is a multi-subunit complex comprising domains V_1_ (600–650 kDa membrane-peripheral domain) and V_0_ (260 kDa membrane-integral domain). The V_1_ domain contains eight different subunits (A–H) and is responsible for ATP hydrolysis, while the V_0_ domain includes six different subunits (a, d, c, c′, c″ and e) and is responsible for proton translocation. There are few reports on the mechanism of V-ATPase regulation in response to various adverse conditions. However, some results have indicated that the expression levels of V-ATPase subunits are involved in various abiotic stresses. For example, overexpression of the wheat (RH8706-49) V-ATPase B subunit (*TaVB*) in *Arabidopsis* conferred higher V-ATPase activity and overall salt tolerance than were observed in the wild type (WT)^16^. Therefore, the cloning and characterization of the V-ATPase subunits may be an effective way to understand the regulation of this molecule and its response mechanism during abiotic stress.

The V-ATPase c subunit (*VHAc*) participates in the formation of a proton channel that is responsible for proton transport and is an essential factor in the assembly of V_1_-V_0_^15^. Mutated yeast lacking *VHAc* fail to assemble V_1_ into the membrane^17^. Some studies have demonstrated the salt regulation capacity of the *VHAc* gene. For example, a transcript analysis of *Pennisetum glaucum PgVHA-c1* showed that the expression of *PgVHA-c1* was increased in response to salinity stress^18^. Under salt stress conditions, overexpression of *LbVHA-c1* (*Limonium bicolor*) in tobacco led to higher activity of superoxide dismutase (SOD) and peroxidase (POD) and lower levels of malondialdehyde (MDA) than in the WT^19^. Despite these findings, there are few reports on gene expression in response to heavy metals, especially related to the *VHAc* gene in a woody halophyte.

We found that V-ATPase activity and ThVHAc1 protein expression in *T. hispida* under CdCl_2_ treatment were much higher than those under NaCl, PEG, or CuSO_4_ treatments (data not shown), indicating that *ThVHAc1* may play a key role in Cd stress tolerance in *T. hispida*. Consistently, we confirmed that the expression of *ThVHAc1* was induced by CdCl_2_ treatment in *T. hispida* roots, stems, and leaves and that the overexpression of *ThVHAc1* in yeast improved Cd tolerance^20^. However, the role of *ThVHAc1* in response to Cd stress and the mechanisms of *ThVHAc1* regulation under Cd stress remain far from being fully elucidated. In this study, we identified a potential upstream regulator of *ThVHAc1*, *ThWRKY7*, which showed expression patterns similar to those of *ThVHAc1* and which improved Cd stress tolerance. In addition, the regulation of plant Cd tolerance by the *ThVHAc1* gene and the relationship between *ThVHAc1* and V-ATPase activity under CdCl_2_ treatment were further analyzed. Our results demonstrate that *ThVHAc1* may participate in Cd tolerance through the reactive oxygen species (ROS) scavenging system to alleviate cell damage. The heterologous expression of *ThVHAc1* effectively improved V-ATPase activity and affected the expression of other subunits and related genes. This study expands our knowledge of the response of *T. hispida* to Cd stress and the relationship of the c subunit with the entire V-ATPase enzyme. Further, our findings provide new insights into the role and regulatory mechanism of *ThVHAc1* upon exposure to Cd stress, which will be beneficial for providing candidate genes to genetically improve tolerance of Cd stress in woody plants.

## Materials and Methods

### Plant materials and treatments

Two-month-old *T. hispida* seedlings were grown in a greenhouse on a 14 h light/10 h dark cycle, with 70–75% relative humidity and an average temperature of 24 °C. The seedlings were well watered at the roots with 150 μM CdCl_2_ for 6, 12, 24, 48, and 72 h, as indicated. Well-watered seedlings were used as the control. The roots, stems and leaves of every treated seedling (sample size of 20 seedlings) were harvested for quantitative real-time PCR (qRT-PCR) analyses. All treatments were applied at least three times (as biological replicates).

### Cloning and expression analysis of the *ThVHAc1* promoter

The *ThVHAc1* promoter was PCR-amplified from *T. hispida* genomic DNA using a genome walking kit (Takara, Japan). The *cis*-elements in the *ThVHAc1* promoter were analyzed using the PLACE database (http://www.dna.affrc.go.jp/PLACE)^21^. The *35S* promoter in pCAMBIA1301 was replaced with the *ThVHAc1* promoter to drive the expression of *β-glucuronidase* (*GUS*) (Fig. S1a), and this construct (named *pThVHAc::GUS*) was transferred into *Arabidopsis*^22^. The T_3_ generation seedlings were used to study the *ThVHAc1* temporal and spatial expression patterns through GUS staining. The 30 d transgenic *Arabidopsis* and *T. hispida* transiently expressing *pThVHAc::GUS* were independently treated with 100 μM CdCl_2_ or H_2_O (as a control) for 0 h, 12 h, or 24 h. Samples were then collected and labeled, and GUS activities were used to analyze the expression of the *ThVHAc1* promoter. The GUS activity was measured according to Hunter and Watson (2008)^23^. When the protein concentrations were high, the samples were diluted with sterile water to maintain A_595_ at less than 2.0. Thus, the determination of GUS activity is precise and avoids the errors generated by saturated staining.

### RNA isolation and qRT-PCR

Total RNA was isolated from each sample using the CTAB method. qRT-PCR was carried out using an MJ Opticon^TM2^ machine (Bio-Rad, Hercules, CA, USA) with the reaction system and procedures from Gao *et al.*(2011)^20^, and *α-Tubulin* (FJ618518), *β-actin* (FJ618517), and *β-tubulin* (FJ618519) were used as internal controls. The primer sequences are listed in Table S1. Relative expression levels were calculated using the ΔΔCt method^24^.

### Identification of the upstream regulator of *ThVHAc1*

The WRKY motif (“GTGACA”) was identified in the *ThVHAc1* promoter (Fig. S1b, S2). A yeast one-hybrid assay was used to find the transcription factors that recognize the WRKY motif. Three tandem copies of the WRKY motif were cloned into a pHis2 vector (pHis2-WRKY) (Fig. S1c). WRKY TFs were identified from seven *T. hispida* libraries and cloned into pGADT7-Rec2 (Clontech, Palo Alto, CA, USA) to produce a cDNA library for use in the one-hybrid assay^25^.

To confirm the interactions between the motif and positive clones, the WRKY core “TGAC” was mutated to “GTCA” and cloned into pHis2 (pHis2-WRKY-M). Fragments of the *ThVHAc1* promoter, including the WRKY motif (pHis2-WRKY-Seg), excluding the WRKY motif (pHis2-WRKY-Seg-M1), or including the mutated WRKY motif (pHis2-WRKY-Seg-M2) were separately cloned into pHis2 (Fig. S1c). The pHis2 plasmid containing three copies of the p53 DNA element (p53His2) was used as a control vector in the yeast one-hybrid assay. All primers are listed in Table S2.

To further confirm the above-mentioned interactions, WRKY, WRKY-M, WRKY-Seg, WRKY-Seg-M1, and WRKY-Seg-M2 were each fused with a *CaMV35S*-46 minimal promoter and cloned into pCAMBIA1301 to drive the *GUS* gene (acting as a reporter) (Fig. S1d). The ORF of *ThWRKY7* (interaction TF) was cloned into the prokII vector under control of the *35S* promoter (prokII-ThWRKY7) (Fig. S1d) to act as an effector. The prokII-ThWRKY7 construct was transferred into *Arabidopsis* using the floral dip method^23^. All reporters were transiently transformed into *ThWRKY7* transgenic *Arabidopsis* using the agrobacterium-mediated transformation method, and all co-transformed *Arabidopsis* leaves were used to measure and stain GUS activity^26^.

### ThVHAc1 transgenic *Arabidopsis*

The ORF of *ThVHAc1* was amplified and cloned into a prokII vector (*35S::ThVHAc1*). The primers are shown in Table S1. *35S::ThVHAc1* was transferred into *Arabidopsis* using the *Agrobacterium*-mediated transformation method^23^. An empty prokII plasmid was also transferred into *Arabidopsis* and used as a control (ck). Kanamycin-resistant lines were detected by PCR using vector-specific primers. The expression level of *ThVHAc1* was confirmed by qRT-PCR, and two transgenic lines with intermediate expression levels (c1#10 and c1#17) were selected for further analysis.

### Stress tolerance analysis

Seeds from the control and T_4_ transgenic *Arabidopsis* were sown on 1/2 Murashige & Skoog (MS) agar medium with 100 μM CdCl_2_. The germination and fresh weight were recorded after 8 d. Six-day-old seedlings sown on 1/2 MS were transferred to 1/2 MS agar plates with an additional 100 μM CdCl_2_ for another 12 d to compare the fresh weight and root length between lines. All experiments were performed three times.

Five-week-old WT and transgenic plants were used to determine the stress tolerance of the transgenic lines. SOD activity, POD activity, glutathione transferase (GST) activity, total chlorophyll content (Tcc), H_2_O_2_ content, proline content, MDA content and electrolyte leakage (EL) were measured after treatment with 100 μM CdCl_2_ for 6 d^27^,^28^. The fresh weights of the aerial parts of seedlings placed on clean filter paper were measured to compare their water loss. The Cd content was determined using atomic florescence spectrometry^29^. Seedlings watered with 1 μM CdCl_2_ for 6 d were used as the control. Leaves sampled from the above lines and treated with 100 μM CdCl_2_ for 0 (control), 1, and 2 h were stained with nitroblue tetrazolium (NBT), 3,3′-diaminobenzidine (DAB), and Evans blue to analyze the *in vivo* accumulation of O^2−^, H_2_O_2_, and cell death in leaves, respectively. ROS produced by intact guard cells and roots were stained with 3 μM 2,7-dichlorofluorescein diacetate (H_2_DCF-DA, Fluka)^30^, and dead cells in the main roots were stained by propidium iodide^31^. ROS and cell death were visualized using a confocal laser-scanning microscope (CLSM) featuring an LSM410 microscope (Zeiss, Jena, Germany) with excitation at 488 nm and emission at 525 nm. Images were acquired using the ZEN 2009 “lite” edition^32^.

Five-week-old transgenic and control *Arabidopsis* treated with 100 μM CdCl_2_ for 0, 3, 6, 9, 12, and 24 h were collected for isolation of total RNA. The expression of 28 V-ATPase subunits, other V-ATPase-related genes, and stress-related genes was examined by RT-PCR or qRT-PCR. The gene names and primer sequences are listed in Table S3.

### Transient expression of *ThVHAc1* in *T. hispida*

*35S::ThVHAc1*, *RNAi::ThVHAc1* and empty Agrobacterium (used as control, labeled as T-ck) were transiently transformed into the aerial parts of five-week-old *T. hispida* seedlings^33^. The transformed seedlings were stained with DAB and Evans blue to visualize the ROS and cell death after treatment with 100 μM CdCl_2_ for 0 (control), 1, or 2 h. *ThVHAc1* expression was analyzed using qRT-PCR. Meanwhile, the SOD, POD, GST, and glutathione peroxidase (GPX) activities, as well as MDA and EL, were assayed. Furthermore, the expression levels of *ThSOD*, *ThPOD*, *ThGSTZ1*, and *ThGPX,* as well as 15 subunits of V*-*ATPase were examined using qRT-PCR. Two other V-ATPase-related genes and five stress response genes were also examined using RT-PCR. The primers are shown in Table S1. Meanwhile, *35S::ThWRKY7* was transiently transformed into *T. hispida* for analysis of Cd tolerance.

### Tonoplast isolation, SDS-PAGE and western blotting

Using a modification of the method of Ma *et al.* (2002)^34^, tonoplasts were isolated from 200 g of aerial tissue from five-week-old transgenic and WT *Arabidopsis* either treated with 100 μM CdCl_2_ or well watered (as a control) for 6 d on a 0–25% sucrose solution plate. Similarly, tonoplasts from *T. hispida* seedlings with transient expression of *35S::ThVHAc1*, empty prokII (named T-ck), or *RNAi::ThVHAc1* treated with 100 μM CdCl_2_ for 0 (control) or 2 h were also isolated. SDS-PAGE of purified V-ATPase (100 μg tonoplast protein) was conducted using 15% polyacrylamide gels according to a previously published procedure^34^,^35^. The blots were performed based on the recommendations of Ma *et al.* (2002)^34^ and Burnette *et al.* (1981)^36^. Meanwhile, the relative activity levels of P-ATPase and F-ATPase from the 25%–50% sucrose solution plate were also tested^34^,^35^.

### Proton pumping assays, ATPase hydrolysis activity, ATPase activity, and protein concentration

A total of 60 μg of the membrane protein from each sample was used to monitor proton pumping activity. The reaction buffer included 10 mM Mes (adjusted to pH 7.5 with Tris), 250 mM sorbitol, 3 mM MgSO_4_, 5 μM acridine orange, and 100 mM KCl, and the reaction was initiated using 2.5 mM MgSO_4_. The ATPase hydrolysis activity was measured by the Pi released from ATPase^34^,^37^. The reaction buffer contained 40 μg protein, 25 mM Mes (adjusted to pH 7.5 with Tris), 50 mM KCl, 3 mM MgSO_4_, 3 mM ATP, inhibitor, and 0.0125 (V/V) Triton X-100. ATPase activity was measured, the reaction buffer included 40 μg protein, 30 mM Tris (adjusted to pH 7.8 with Mes), 50 mM KCl, 0.5 mM MgSO_4_, 0.3 mM PPi-Tris (pH = 7.8), and inhibitor. Protein concentrations were estimated as described by Lowry *et al.* (1951)^38^. The inhibitors used for determining V-ATPase-, P-ATPase-, and F-ATPase-related activities were NaN_3_ (0.6) + Na3VO_3_ (0.6), NaNO_3_ (50) + NaN_3_ (0.6), and NaNO_3_ (50) + Na_3_VO_3_ (0.6) [mM], respectively. All experiments were performed at least three times.

## Results and Discussion

### Cloning and analysis of *ThVHAc1* promoter

**Figure 1:**
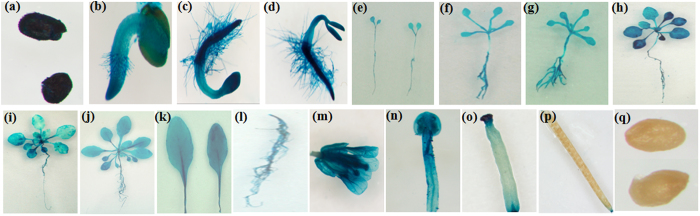
Spatiotemporal characterization of *ThVHAc1* by the analysis of *ThVHAc1* promoter activity in *pThVHAc::GUS* transgenic *Arabidopsis*. (**a–q**) Expression of *ThVHAc1* at different growth stages and in different organs or tissues: (**a**) dry seed; (**b**) 2-d-old bud; (**c**) 3-d-old bud; (**d**) 4-d-old bud; (**e**) 6-d-old seedling; (**f**) 10-d-old seedling; (**g**) 16-d-old seedling; (**h**) 22-d-old seedling; (**i**) 28-d-old seedling; (**j**) 34-d-old seedling; (**k**) leaves of 34-d-old seedling; (**l**) roots of 34-d-old seedling; (**m**) whole flower cluster; (**n**) stigma; (**o**) bracteole; (**p**) intact fresh silique and its seeds; (**q**) immature seeds.

**Figure 2:**
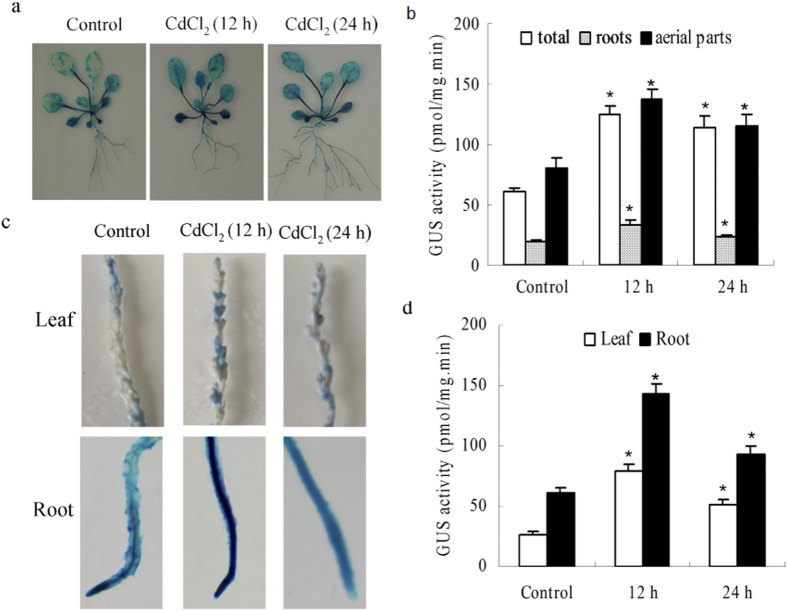
The GUS staining expression analysis in *pThVHAc*::GUS-transformed *Arabidopsis* after CdCl_2_ treatment and transient expression in *T. hispida*. (**a**) 30-d-old transformed *Arabidopsis* under normal conditions and CdCl_2_ treatment. (**b**) The GUS activity according to a. (**c**) GUS staining of *T. hispida* transiently transformed with *pThVHAc*::GUS. (**d**) The GUS activity according to (**c**) Data are shown as the mean ± SD. The asterisks in (**b**,**d**) indicate significant differences between treatment and control (P < 0.05).

A 1,164 bp promoter fragment (from −1 to −1164) was amplified by TAIL-PCR, and *pThVHAc*::GUS transgenic *Arabidopsis* was generated. GUS staining in *Arabidopsis* revealed GUS activity in mature seeds, cotyledons, leaves, stems, roots, petals, stamen, stigma, pistils, and anthers but not in fresh pods or immature seeds (Fig. 1a–q), indicating that *ThVHAc1* expression is tissue-specific. Consistent with this finding, Padmanaban *et al.*^39^ (2004) detected strong GUS activity in the root cap in *AtVHA-c3* promoter::GUS transgenic plants, and *AtVHA-c3* dsRNA-mediated mutant lines exhibited decreased root length and diminished salt tolerance. These results indicated that the roots play a role in the regulation of abiotic stresses, especially Cd tolerance, by *ThVHAc1*. Furthermore, GUS activity was more obvious in roots and leaves than in stems (Fig. 1a–l). Upon exposure to CdCl_2_ for 12 h, the GUS activity increased 2.04-fold in aerial parts and 1.77-fold in roots compared to control conditions. When treated for 24 h, the increase was 1.87-fold in aerial parts and 1.22-fold in roots (Fig. 2a,b), indicating that the *ThVHAc1* promoter confers a tissue-specific Cd stress response. Furthermore, GUS staining also revealed the transient expression of *pThVHAc*::GUS in *T. hispida*, showing that the GUS activity increased after CdCl_2_ treatment. The GUS activity increased 2.99-fold or 1.94-fold in leaves and 2.34-fold or 1.51-fold in roots when treated with CdCl_2_ for 12 or 24 h, respectively (Fig. 2c,d). All GUS activities in both transgenic *Arabidopsis* and transgenic *T. hispida* increased after CdCl_2_ treatment for 12 h and 24 h, and these activity levels were consistent with the transcription level of *ThVHAc1* in *T. hispida* roots and leaves under the same treatments, indicating a correlation between the promoter activity and *ThVHAc1* gene expression pattern in plants exposed to Cd stress.

A PLACE database (http://www.dna.affrc.go.jp/PLACE) comparison of the *ThVHAc1* promoter revealed many abiotic stress-related *cis*-elements, such as ARR1AT, CAATBOX, DOFCOREZM, EBOXBNNAPA, GT1CONSENSUS, MYCCONSENSUSAT, NODCON1GM, WBOX, and WRKY710S (Fig. S2), indicating that *ThVHAc1* may be regulated by different types of transcription factors (TFs). In particular, the promoter contains eighteen WBOX and WRKY motifs that are found in many promoters of stress tolerance genes, such as *TaeIF5A*^40^, *PROPEP2* and *PROPEP3*^41^, and *GbDXS* and *GbGGPPS*^42^. The promoter of pathogenesis-related protein (PR) in parsley also contains WRKY motifs to which *WRKY* transcript factors specifically bind, and these motifs were further used to identify PR proteins^43^. In bananas, ethylene-induced ripening induces the expression of both the PR gene and the *V-ATPase c”* subunit in fruit tissue, suggesting a probable interaction between the PR and *V-ATPase c”* genes^44^. Thus, *WRKY* genes may also regulate *V-ATPase c”* gene expression. Moreover, the V-ATPase c” subunit shares high homology with VHA-c, and their expression patterns under different stresses were highly similar in *T. hispida* roots, stems, and leaves (data not shown), suggesting a possible synergistic function in stress tolerance. These results led us to investigate whether WRKY motif-binding TFs bind specifically to the *ThVHAc1* promoter and function as its upstream regulator to control *ThVHAc1* expression during stress tolerance.

### *ThWRKY7* is an upstream regulator of *ThVHAc1*

**Figure 3:**
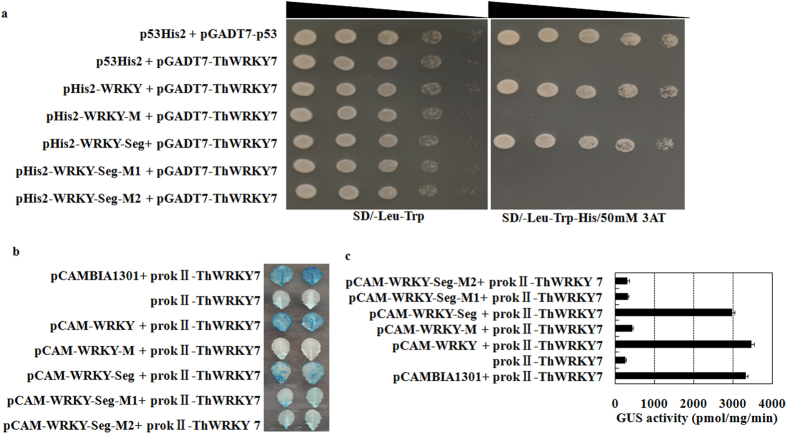
Yeast one-hybrid analyses of the upstream regulators of *ThVHAc1*. (**a**) The clones on SD/-Leu-Trp were used as positive controls, further confirmed by spotting serial dilutions (1/1, 1/10, 1/100, 1/1000) onto SD/-His/-Leu/-Trp plates with 50 mM 3-AT, and the triangle indicates the dilutions from 1/10 to 1/1000. (**b**) The effect of the overexpression of ThWRKY7 in *Arabidopsis* on the transiently expressed reporter. M, mutated WRKY motif. M1, *ThVHAc1* promoter fragment without the WRKY motif. M2, *ThVHAc1* promoter fragment containing the mutated WRKY motif. (**c**) GUS activity according to (**b**).

A yeast one-hybrid assay was used to verify the interaction between TFs and the WRKY motif in the promoter. *ThWRKY7* was found to bind to the WRKY motif, as shown by the interaction between pHis2-WRKY-Seg and *ThWRKY7* and the absence of an interaction between pHis2-WRKY-M/M1/M2 and *ThWRKY7* on the SD/-Trp-Leu-His/50 mM 3-AT (3-amino-1, 2, 4-triazole) solid medium (Fig. 3a). Furthermore, the effector construct prokII-*ThWRKY7* was transferred into *Arabidopsis*, and T_3_ seedlings were used to detect transient expression of reporter plasmids. The reporter plasmids were constructed in pCAMBIA1301 harboring the intact or mutated WRKY motif, harboring the *ThVHAc1* promoter fragment with its intact or mutated WRKY motif, or without the WRKY motif, followed by a 46 bp minimal promoter. The leaves of prokII-*ThWRKY7* transgenic T_3_ seedlings were then transiently transformed with one of the above-mentioned reporter plasmids. GUS activity was clearly detected in prokII-*ThWRKY7* transgenic leaves with the reporter plasmid containing the *ThVHAc1* promoter fragment with its intact WRKY motif, whereas other mutated reporters did not activate GUS expression (Fig. 3b,c). These results further demonstrate that *ThWRKY7* may bind specifically to the WRKY motif in the *ThVHAc1* promoter.

**Figure 4:**
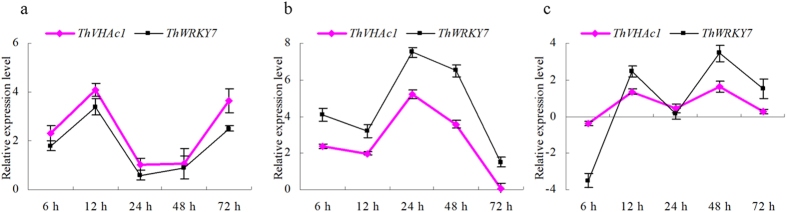
The expression patterns of *ThVHAc1* and *ThWRKY7* under CdCl_2_ treatment. (**a**) Roots. (**b**) Stems. (**c**) Leaves. The relative expression levels were all log_2_ transformed. All experiments were repeated three times. The data are shown as the means ± SD of three independent experiments.

qRT-PCR analysis of *ThVHAc1* and *ThWRKY7* showed that the two genes displayed similar expression patterns under CdCl_2_ treatment. Specifically, the relative expression levels of both genes in roots increased before 12 h after CdCl_2_ treatment and then decreased, reaching their lowest levels at 24 or 48 h (Fig. 4a). In stems, the expression of *ThWRKY7* was slightly higher than that of *ThVHAc1* at every time point. However, the two genes exhibited the same expression pattern, with the expression levels being highest at 24 h and lowest at 72 h (Fig. 4b). In leaves, the two genes were downregulated at 6 h and exhibited peak expression at 48 h (Fig. 4c). This synchronized expression patterns indicate that *ThWRKY7* may play a critical role in either regulating the *ThVHAc1* expression or cooperating with *ThVHAc1* to improve plant Cd stress tolerance. To further confirm this conclusion, *ThWRKY7* was transiently overexpressed in *T. hispida*. The leaves transformed with *35S::ThWRKY7* showed lower levels of DAB and Evans blue staining as well as slower accumulation of MDA and EL than did leaves transformed with T-ck. Moreover, the SOD, POD, GST, and GPX activities of leaves transformed with *35S::ThWRKY7* were significantly higher than those of leaves transformed with T-ck (Fig. S3), suggesting that overexpression of *ThWRKY7* also markedly improves Cd tolerance, further demonstrating that *ThWRKY7* may fine-tune the *ThVHAc1*-mediated Cd stress response.

*WRKY* transcription factors are a complex family with previously reported relationships to the plant immune response, in which they function as either positive or negative regulators^45^,^46^. Various functions in the protection process are a basic feature of *WRKY* genes, and the redundant elements in the promoters of their target genes imply a regulatory capacity of *WRKY*^47^. *WRKY* TFs regulate plant tolerance to abiotic stress by binding to the *WRKY cis*-element present in many stress-related and co-regulated gene promoters in *Arabidopsis*^46^. Regarding our results, the binding of *ThWRKY7* to the WRKY element in the *ThVHAc1* promoter, as well as the similar expression profiles of these elements over time when exposed to Cd stress, suggest that *ThVHAc1* and *ThWRKY7* may co-regulate Cd tolerance and that *ThWRKY7* may control *ThVHAc1* in the improvement of abiotic stress tolerance.

### Heterologous expression of *ThVHAc1* improves Cd tolerance in *Arabidopsis*

**Figure 5:**
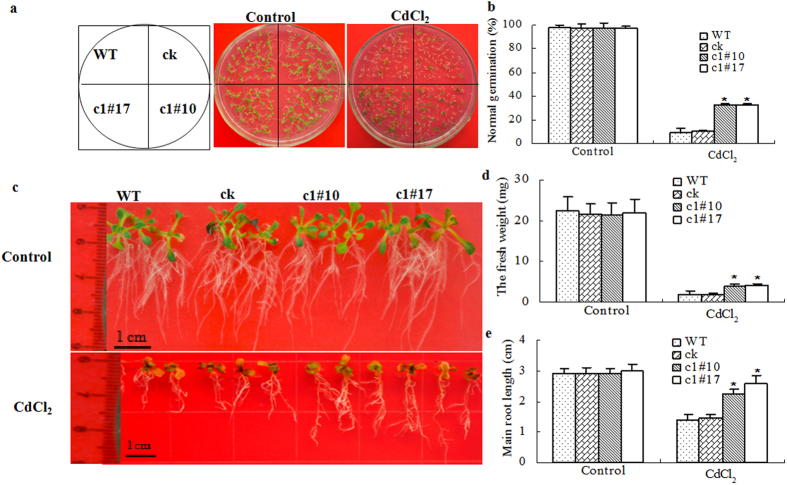
Cd tolerance assay of wild type (WT), empty prokII-transformed line (control, marked as ck), and transgenic *Arabidopsis* c1#10 and c1#17. (**a**) Germination under 100 μM CdCl_2_ treatment for 8 days. (**b**) Germination percentage according to (**a**). (**c**) 6-day-old seedlings of WT, ck, c1#10, and c1#17 grown under normal conditions (1/2 MS medium) were transferred to medium supplemented with 100 μM CdCl_2_ for another 12 d. (**d**,**e**) Fresh weight and root length of the transgenic *Arabidopsis* under Cd stress. The all experiments were repeated three times, and thirty *Arabidopsis* seedlings were used for each treatment. All data are shown as the mean ± SD. The asterisks in (**b**,**d**,**e**) indicate significant differences between transgenic lines and WT (P < 0.05).

To study the function of *ThVHAc1* in plants, *ThVHAc1* was overexpressed in *Arabidopsis*, and two transgenic lines (c1#10 and c1#17) were subjected to CdCl_2_ treatment. The results showed that the germination, fresh weight, and main root length did not differ among c1#10, c1#17, WT, and ck under normal conditions (Fig. 5). However, when exposed to the CdCl_2_ treatment c1#10 and c1#17 showed better biomass accumulation than WT and ck. The average germination, fresh weight, and main root length of c1#10 and c1#17 were 3.3-, 1.5-, and 1.7-fold greater, respectively, than those of both WT and ck (P < 0.05) (Fig. 5), indicating that the heterologous expression of *ThVHAc1* can improve biomass and germination of *Arabidopsis* under Cd stress.

**Figure 6:**
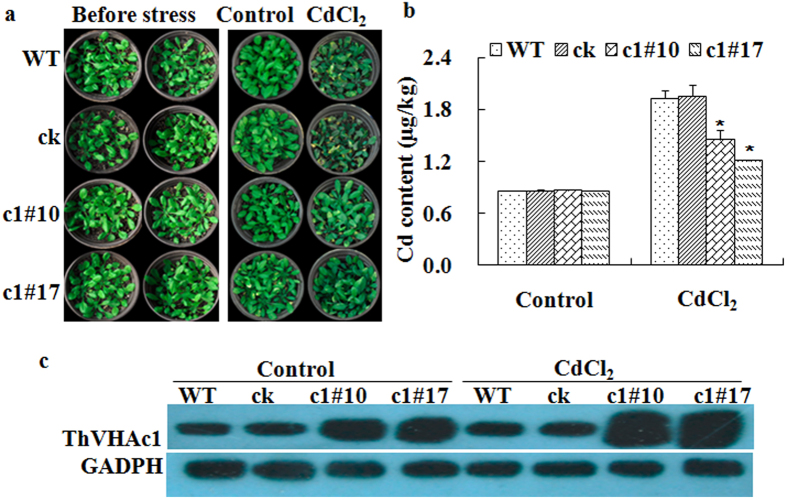
Cd stress tolerance analysis in soil growth conditions. (**a**) Growth states of the four lines before and after treatment. (**b**) Cd content comparison between control lines and *ThVHAc1* transgenic seedlings after CdCl_2_ treatment. (**c**) Western blotting analysis of tonoplast V-ATPase, using antibodies against the ORF full-length protein synthesized from *ThVHAc1* (Abmart, Inc., Shanghai, China) and AHA3 (bs-2247R).

The Cd content was determined in two transgenic and two control lines. Under the control condition, the levels of Cd accumulation did not differ among the four lines, but when treated with 100 μM CdCl_2_, the Cd content in WT and ck was an average of 1.4-fold higher than that in c1#10 and c1#17, which is a statistically significant difference (Fig. 6a,b). This evidence suggests that the heterologous expression of *ThVHAc1* can reduce Cd accumulation in plants.

**Figure 7:**
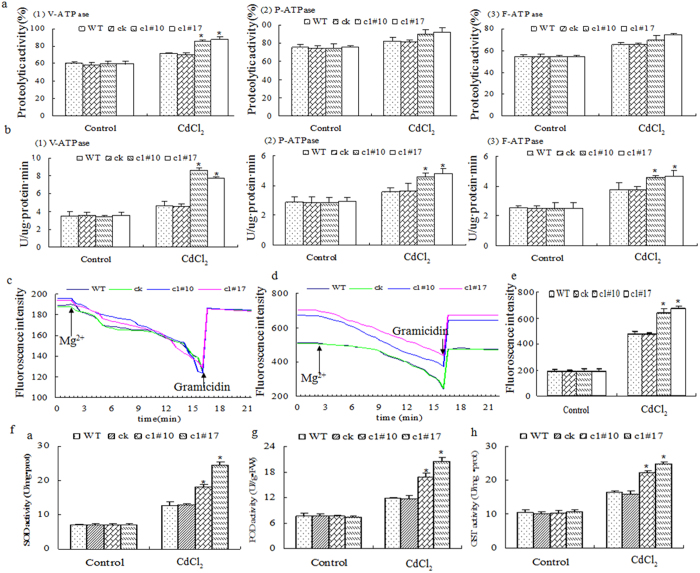
V-ATPase activity and other related enzymes activities analysis of five-week-old *Arabidopsis* with heterologous expression of *ThVHAc1* and treated with CdCl_2_ for 6 d. V-ATPase-related activities were measured under the addition of the inhibitor 0.6 mM NaN_3_ + Na_3_VO_3_. P-ATPase-related activity was measured under the addition of the inhibitor 0.6 mM NaN_3_ + 50 mM NaNO_3_. F-ATPase-related activity was measured under the addition of the inhibitor 50 mM NaNO_3_ + 0.6 mM Na_3_VO_3_. (**a**), Hydrolytic activity. (**b**) ATPase activity. (**c**) Proton transport activity under control. Gramicidin D was used to collapse the residual pH gradient. (**d**) Proton transport activity under Cd stress. (**e**) The proton transport activity according to (**c,d**). Proton transport activity was evaluated by fluorescence quenching as measured by a Hitachi 4010 fluorescence spectrophotometer with 495 nm excitation and 525 nm emission. The reaction was started with MgSO_4_, after equilibrium, with 4 mM gramicidin D to collapse the pH gradient. The equilibrium fluorescence quenching after treatment with 4 mM gramicidin D. (**f**) SOD activity. (**g**) POD activity. (**h**) GST activity. Data are shown as the mean ± SD. The asterisks indicate significant differences between transgenic lines and WT (P < 0.05).

Heavy metal stress always leads to the production of ROS and to disturbed cellular redox status. Plants respond by increasing the production of a series of enzymes such as V-ATPase, SOD, POD, GPX, ascorbate peroxidase (APX), and GST^48^,^49^. Therefore, we compared the V-ATPase activity between the control and *ThVHAc1* transgenic lines under CdCl_2_ treatment for 6 d. The tonoplasts of WT, ck, and transgenic lines were isolated. Western blotting indicated the successful isolation of tonoplast, as determined by the detection of V-ATPase (Fig. 6c). Assays for V-ATPase-related activities were performed, and all four *Arabidopsis* lines displayed similar hydrolytic activity, ATPase activity, and proton transport activity under normal conditions. After CdCl_2_ treatment, all V-ATPase-related activities were increased in all lines, but the activities in the transgenic *ThVHAc1* lines were increased more than those in the control WT and ck lines. The average V-ATPase activities, hydrolytic activities, and proton transport activities of c1#10 and c1#17 were 1.8-, 1.2-, and 1.4-fold greater than those of the control lines, respectively (Fig. 7). These results indicate that the heterologous expression of *ThVHAc1* leads to an increase in V-ATPase-related activities in response to Cd stress.

Similarly to V-ATPase, F-ATPase and P-ATPase may also be involved in stress tolerance. Lemos *et al.* (2005) showed that F-ATPase functions in maintaining cytoplasmic pH, determining the acid tolerance of cariogenic *Streptococci mutans*^50^. P-ATPase was previously shown to be an important factor in salt tolerance^48^,^51^,^52^. In this study, the activity of F-ATPase and P-ATPase in isolated tonoplast samples was measured after the addition of the corresponding inhibitors, and the results were very similar to those for V-ATPase activity (Fig. 7). Compared with WT and ck, the activity of F-ATPase and P-ATPase were increased 1.2- and 1.3-fold, respectively, in *Arabidopsis* lines overexpressing *ThVHAc1*. Taken together, this finding demonstrates that the regulation of Cd tolerance is complex, and the possible roles of F-ATPase and P-ATPase in Cd tolerance merit further study.

The activity of the antioxidants SOD, POD and GST also did not differ among the four lines under normal conditions. After exposure to CdCl_2_ treatment, the SOD, POD, and GST activities in c1#10 and c1#17 were significantly higher than those in WT and ck. The SOD activity of c1#17 was 1.92-fold greater than that of WT, the POD activity of c1#17 was 1.77-fold greater than that of the control lines, and the average GST of the transgenic lines was 1.46-fold greater than that of the control lines (Fig. 7f–h). These results suggested that the heterologous expression of *ThVHAc1* correlates with increased activities of protective regulatory enzymes under Cd stress. A previous study suggested that antioxidant activity, such as that of POD in *Kandelia candel* and lipid peroxidation in *Bruguiera gymnorrhiza*, can be used as a biomarker for heavy metal stress conditions^53^. In the current study, the activities of V-ATPase, SOD, POD, and GST were all increased and higher than the corresponding activities in the control lines exposed to Cd stress, indicating that the heterologous expression of *ThVHAc1* increased the activities of the above enzymes, keeping the ROS level low in transgenic *Arabidopsis*.

**Figure 8:**
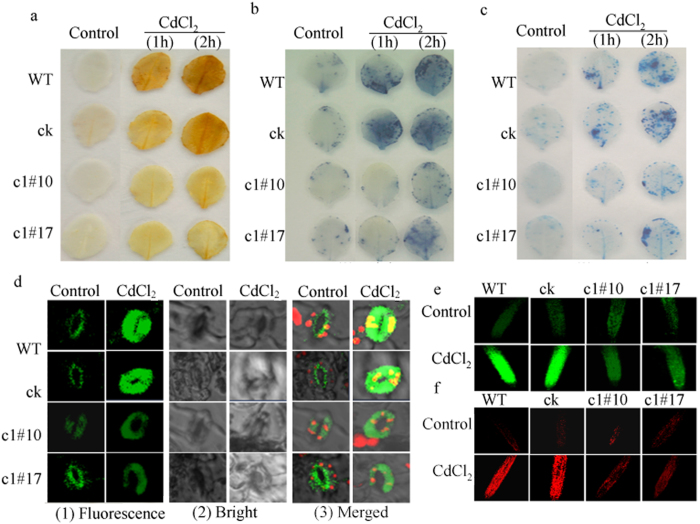
ROS accumulation and cell damage in WT, ck, c1#10, and c1#17 under 100 μM CdCl_2_ treatment. All experiments were repeated at least three times, and approximately 15 leaves collected from multiple seedlings at five weeks old were inspected in each experiment. (**a**) DAB staining. (**b**) NBT staining. (**c**) Evans blue staining. (**d**) ROS production in intact guard cells is indicated by the fluorescent dye DCF after exposure to 2 h stress. Epidermal peels were loaded with H_2_DCF-DA for 10 min after the incubation for 2 h. (**e**) ROS production in root indicated by the fluorescent dye DCF, consistent with d. Main roots were incubated in incubation buffer for 2 h at room temperature and then stained with 5 μM D H_2_DCF-DA for 10 min. (**f**) Cell damage of main roots stained by 5 μM propidium iodide; the treatment condition were as described in (**d**).

**Figure 9:**
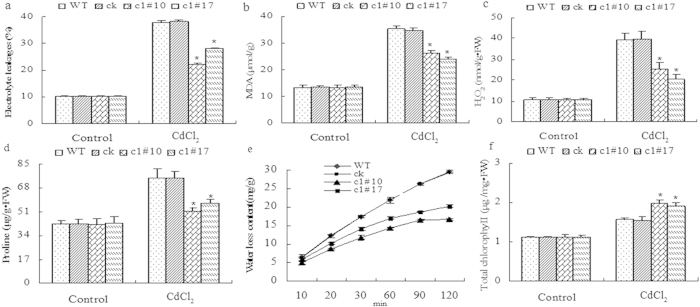
Physiological index analyses of five-week-old seedlings of WT, ck, c1#10, and c1#17 under 100 μM CdCl_2_ for 6 d. Well-watered seedlings were used as controls. (**a**) Electrolyte leakage. (**b**) MDA content. (**c**) H_2_O_2_ content. (**d**) Proline content. (**e**) Weight of water loss. (**f**) Total chlorophyll. All experiments were repeated three times. The data are the means ± SD of three independent experiments. All data are shown as the mean ± SD. The asterisks indicate significant differences between transgenic lines and WT (P < 0.05).

Consistent with the above results, the histochemical staining of the ROS level showed that under normal conditions, levels of DAB staining for H_2_O_2_ and NBT staining for O^2−^ in leaves were similar among WT, ck, c1#10, and c1#17. After CdCl_2_ treatment, c1#10 and c1#17 accumulated less H_2_O_2_ and O^2−^ than did WT and ck (Fig. 8a,b). The H_2_DCF staining of ROS in intact guard cells and main roots also showed that ROS accumulation in WT and ck was higher than that in c1#10 and c1#17 under CdCl_2_ treatment (Fig. 8d,e), suggesting a positive role for *ThVHAc1* in regulating the ROS level in plants under Cd stress. In addition, Evans blue and propidium iodide staining for cell damage in leaves and main roots also revealed less cell damage in transgenic lines than in WT and ck (Fig. 8c,f). Meanwhile, the EL rate and MDA, H_2_O_2_ and proline contents of WT and ck were also significantly higher than those of c1#10 and c1#17 (p < 0.05) (Fig. 9a–d), confirming that the Cd stress response involves ROS metabolism and that *ThVHAc1* may play a positive role in Cd stress tolerance by controlling ROS homeostasis.

Plants exposed to various stresses need to maintain normal metabolic functions, such as growth^54^, water-holding capacity^55^ and chlorophyll content^56^. Water and chlorophyll are requirements for photosynthesis. Our results showed that the transgenic lines had higher water-holding capacity than the control lines (Fig. 9e) and that after CdCl_2_ treatment, the chlorophyll content of c1#10 and c1#17 was higher than that of WT and ck (Fig. 9f). Figure 6a also shows that growth for all four lines is similar under normal conditions; however, after exposure to CdCl_2_ treatment for 6 d, c1#10 and c1#17 displayed greener leaves than WT and ck. All these results suggest that the heterologous expression of *ThVHAc1* in *Arabidopsis* increased the activity of both V-ATPase and antioxidants, which may regulate ROS homeostasis, cell damage, and photosynthesis for better Cd stress tolerance.

### Cd tolerance analysis in *T. hispida* transiently expressing *ThVHAc1*

**Figure 10:**
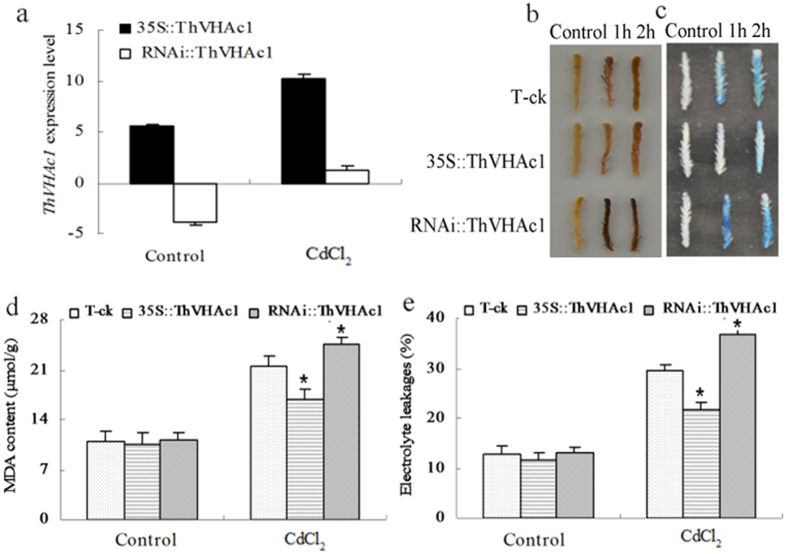
Analysis of transient expression of *ThVHAc1* in *T. hispida* under 100 μM CdCl_2_ treatment for 1 h and 2 h. (**a**) qRT-PCR analysis of *ThVHAc1* in *T. hispida* seedlings transiently transformed with *35S::ThVHAc1,* RNAi::*ThVHAc1* compared with T-ck. The relative expression levels were all log_2_ transformed. (**b**) DAB staining. (**c**) Evans blue staining. (**d**) MDA content. (**e**) Electrolyte leakage. All data are displayed as the mean ± SD of three independent experiments, and significant differences between transgenic lines and WT (P < 0.05) are indicated by asterisks.

To further confirm the results of the heterologous expression of *ThVHAc1* in *Arabidopsis*, the overexpression vector *35S::ThVHAc1*, the suppression expression vector *RNAi::ThVHAc1* and the empty vector T-ck were transiently expressed in *T. hispida*. qRT-PCR results revealed an expression level of *ThVHAc1* in *35S::ThVHAc1* that was 47.17-fold greater than that of T-ck, and the expression of *RNAi::ThVHAc1* was only 6.79% that of T-ck (Fig. 10a), indicating that the transient expression in these lines was successful.

**Figure 11:**
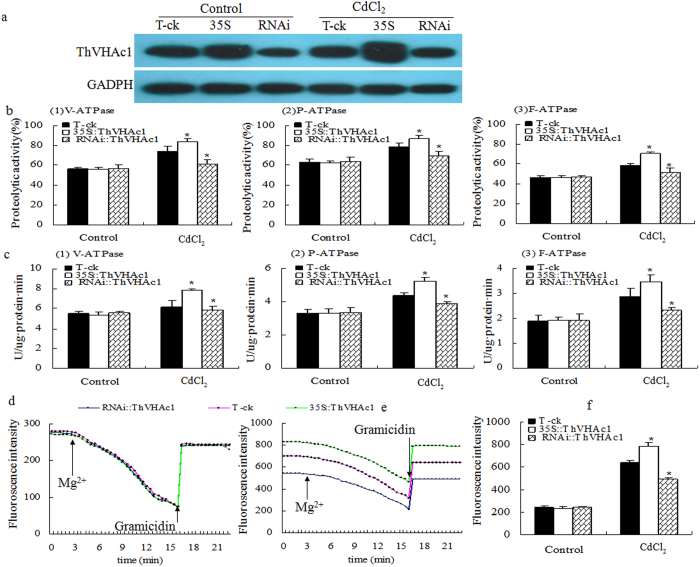
V-ATPase activity analysis of transient expression of *ThVHAc1* in *T. hispida* under CdCl_2_ treatment for 2 h. V-ATPase related activities were measured as those in *Arabidopsis*. (**a**) Western blotting analysis of tonoplast V-ATPase. (**b**) Hydrolytic activity. (**c**) ATPase activity. (**d**) Proton transport activity under control. (**e**) Proton transport activity under Cd stress. (**f**) Proton transport activity according to (**d**,**e**). All data are displayed as the mean ± SD, and significant differences (P < 0.05) are indicated by asterisks.

DAB and Evans blue staining of these transient expression lines showed that the ROS accumulation in *RNAi::ThVHAc1* was higher than that in T-ck, while the lowest ROS accumulation was observed in *35S::ThVHAc1* under CdCl_2_ treatment (Fig. 10b,c). The EL and MDA levels in *35S::ThVHAc1* were also significantly lower than those in T-ck and *RNAi::ThVHAc1*. Specifically, the EL in *35S::ThVHAc1* was 73.6% of that in T-ck and 58.9% of that in *RNAi::ThVHAc1*, and the MDA content in *35S::ThVHAc1* was 78.2% of that in T-ck and 69.0% of that in and *RNAi::ThVHAc1* (Fig. 10d,e). The tonoplasts of these lines were isolated, as confirmed by western blotting (Fig. 11a), and all control, *RNAi::ThVHAc1*, and *35S::ThVHAc1* lines showed similar V-ATPase activities before Cd stress. However, after treatment with CdCl_2_, *35S::ThVHAc1* displayed the highest V-ATPase activity and *RNAi::ThVHAc1* the lowest. The V-ATPase activity, hydrolytic activity and proton transport activity of *35S::ThVHAc1* were 1.3-, 1.4-, and 1.6-fold greater than those in the *RNAi::ThVHAc1* line, respectively (Fig. 11). The F-ATPase and P-ATPase activities showed tendencies similar to that of V-ATPase activity. In *35S::ThVHAc1*, the F-ATPase and P-ATPase activities were 1.5- and 1.4-fold greater than those in *RNAi::ThVHAc1*, respectively, while the corresponding hydrolytic activities were 1.4- and 1.2-fold greater than those in *RNAi::ThVHAc1* (Fig. 11).

**Figure 12:**
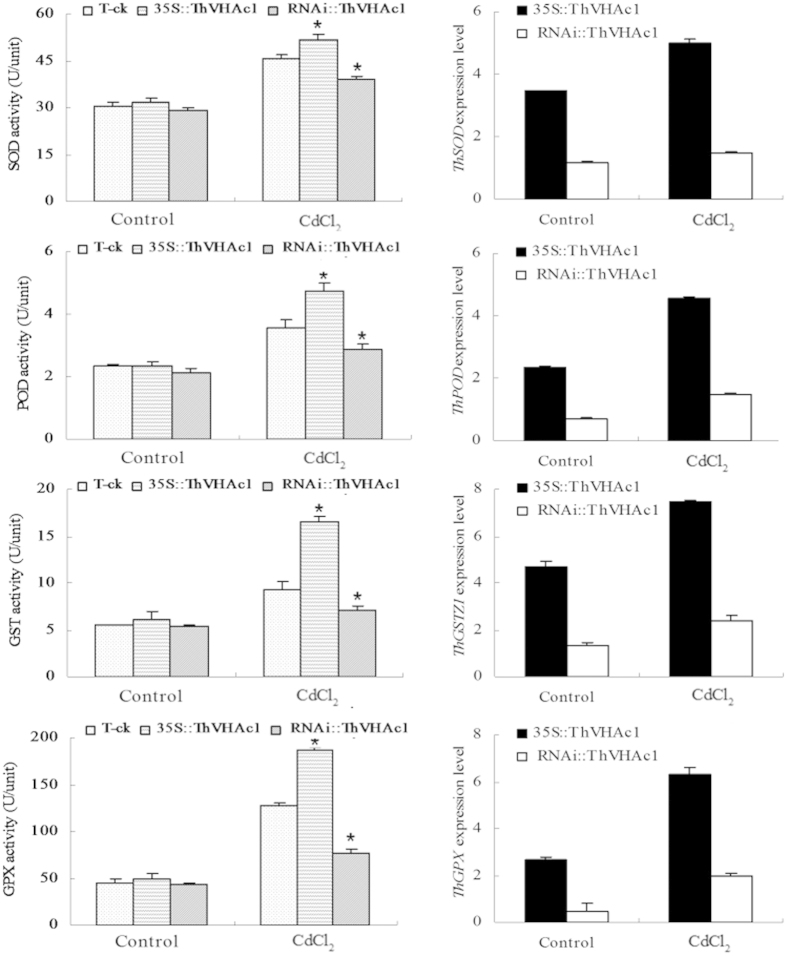
The SOD, POD, GST, GPX activities, and the expression of *ThSOD*, *ThPOD*, *ThGSTZ1*, *ThGPX* analysis of T-ck, *35S::ThVHAc1,* RNAi::*ThVHAc1* seedlings. All data are displayed as the mean ± SD of three independent experiments, and significant differences between transgenic lines and WT (P < 0.05) are indicated by asterisks.

Furthermore, the activities of protective enzymes, including SOD, POD, GST, and GPX, were significantly higher in *35S::ThVHAc1* than in *RNAi::ThVHAc1* and T-ck after CdCl_2_ treatment (Fig. 12). These results further suggest that *ThVHAc1* participated in the regulation of Cd tolerance by increasing the activity of protective enzymes to maintain ROS homeostasis in cells. Taken together, these results indicate that *ThVHAc1* may be an effective gene for improving plants’ Cd tolerance.

### Expression of *ThVHAc1* influenced other related genes and V-ATPase subunits

To investigate whether other genes were affected by the expression of *ThVHAc1,* the expression levels of five *AHA* (H^+^-ATPase), five *ACA* (auto-inhibited Ca^2+^-ATPase), and eight stress-related genes were analyzed by RT-PCR. *AHA* genes primarily participate in ATP binding, the biosynthetic process, protein phosphorylation-dependent regulation, and coupling with transmembrane ion movement^57^. One of the *AHA* genes, *At3g42640*, was reported to be induced during *Arabidopsis* pollen development and during fertilization in *B. campestris* subsp*. Chinensis*^58^. *ACA* genes function in ATP activity, calcium channel activity, catalytic activity, hydrolase activity, and carbonate dehydratase activity^59^. When exposed to a boron deficiency for 24 h, the transcriptional level of the *ACA* gene *At1g27770* increased by 1.43-fold^60^. *CSD* (cytosolic copper/zinc superoxide dismutase, At1g08830) is involved in ROS accumulation^61^, and *APX* (ascorbate peroxidase, At1g07890) is an ascorbate peroxidase with increased activity under oxidative stress in DET2-mutant *Arabidopsis*^62^. The transcription levels of *RBOHC* (respiratory burst oxidase homolog c, At5g51060) and *LOX1* (lipoxygenase, At3g45140) were markedly upregulated when *Arabidopsis* was exposed to Cd stress^63^.

**Figure 13:**
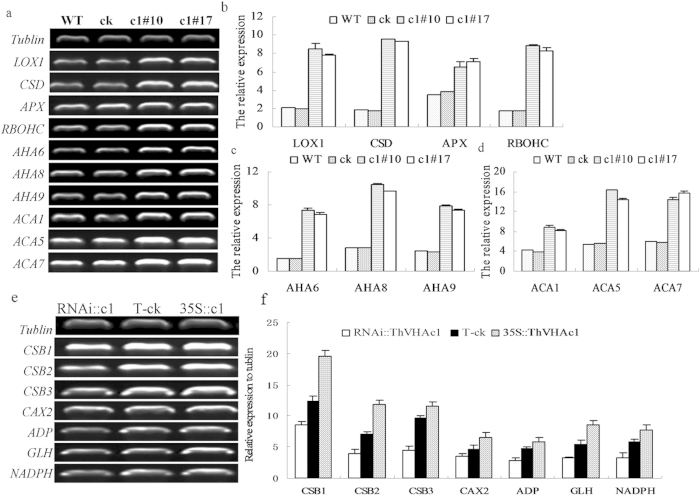
RT-PCR analysis of stress-related genes in *Arabidopsis* with heterologous expression of *ThVHAc1* and in *T. hispida* with transient expression of *ThVHAc1*. (**a**) Gel electrophoresis of *AHA* genes (At2g07560, At1g80660, At3g42640), *ACA* genes (At1g27770, At1g08065, At1g08080) and stress-related genes (*ACT* (At3g18780), *CSD* (At1g08830), *APX* (At1g07890), *RBOHC* (At5g51060)). (**b–d**) Relative expression levels according to a. (**e**) Gel electrophoresis of *CSB1-3*, *CAX2*, *ADP*, *GLH*, *NADPH*. (**f**) Relative expression levels according to (e). All experiments were repeated three times. The data are shown as the means ± SD of three independent experiments.

The results of the present study revealed that three *AHA* genes (At2g07560, At1g80660, and At3g42640), three *ACA* genes (At1g27770, At1g08065, and At1g08080) and four stress-related genes [(*LOX1* (At3g45140), *CSD* (At1g08830), *APX* (At1g07890), and *RBOHC* (At5g51060)] were expressed at higher levels in the transgenic *Arabidopsis* c1#10 and c1#17 lines than in the WT and ck lines. For example, the *ThVHAc1* transgenic lines expressed the *AHA* genes at levels more than 3-fold those of the WT. The highest expression level of the *AHA* gene was approximately 4.9-fold higher (relative to ck) in c1#10, while that of the *CSD* gene was 5.3-fold higher (relative to ck) in c1#10 (Fig. 13a–d).

In three transiently transformed *T. hispida* lines, four antioxidant genes were analyzed using qRT-PCR. *ThSOD*, *ThPOD*, *ThGSTZ1* and *ThGPX* showed similar expression profiles, all of which were upregulated after CdCl_2_ treatment. The expression levels of these genes were highest in *35S::ThVHAc1* and lowest in *RNAi::ThVHAc1* (Fig. 12). We also characterized the expression of several genes associated with stress-related functions and V-ATPase activity in *T. hispida*. The upregulated genes are shown in Fig. 13e,f, including one vacuolar cation/proton exchanger isoform *CAX2* gene, three chloroplast protease genes (*CSB*), one ATP-dependent protease proteolytic subunit (*ADP*), one glycoside hydrolase protein (*GLH*) and one *NADPH* gene. These results indicate that the expression of *ThVHAc1* changed the expression of other stress-related and V-ATPase-related genes, suggesting a complex network of Cd tolerance regulation.

Consistent with this result, other researchers have also shown that the overexpression or suppression of some genes always affects other genes. For example, overexpression of a *DREB* gene affected the expression of *SOD*, *GST*, and other stress-related genes^27^. R740S mutation in the a3 subunit of V-ATPase decreased the expression of key osteoclast markers (TRAP, cathepsin K, OSCAR, DC-STAMP, and NFATc1)^64^. The overexpression of *SaVHAc1* in rice upregulated many stress-related genes, such as cysteine synthase, the pathogenesis-related protein Bet vI family protein, and glutamine synthetase under salt stress^65^.

**Figure 14:**
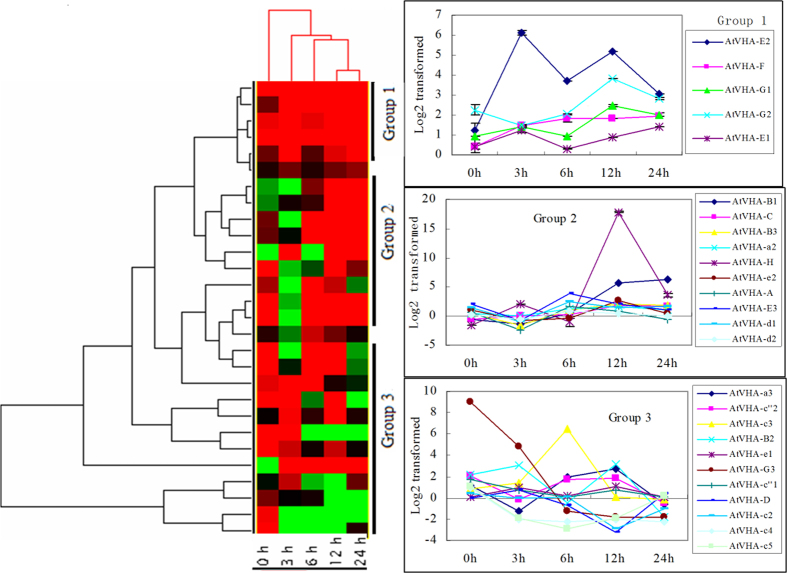
Cluster analysis of the expression levels of all subunits in c1#10 based on WT. The subunits were classified into three groups. The log2-transformed expression levels were calculated according to the three groups. The x-axis shows the stress time point. The data are shown as the means ± SD of three independent experiments.

**Figure 15:**
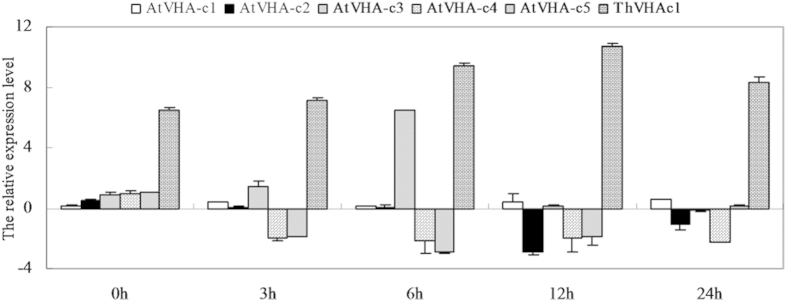
qRT-PCR analysis and comparison of five *AtVHA-c* subunits and *ThVHAc1* in c1#10. The relative expression levels were all log_2_ transformed. The x-axis shows the stress time point. The data are shown as the means ± SD of three independent experiments.

V-ATPase is a multi-subunit enzyme. Overexpression of the *ThVHAc1* gene affected many aspects of V-ATPase activity under CdCl_2_ treatment (Figs 7 and 11). To better understand whether other subunits of V-ATPase were also affected by the expression of *ThVHAc1* under CdCl_2_ treatment, the expression profiles of 28 subunits in *Arabidopsis* were analyzed by qRT-PCR in c1#10 and c1#17 at different times. Clustering analysis of the expression patterns of all 28 subunits in c1#10 showed that they were primarily clustered into three groups. All subunits in group 1, including AtVHA-E2, F, G1, G2 and E1, were upregulated. Meanwhile, most subunits in group 2, including AtVHA-B1, C, B3, a2, H, e2, A, E3, d1 and d2, were induced after 12 h of treatment. The remaining subunits, except a1 and c1, belong to group 3 and were primarily suppressed, especially after 24 h of treatment (Fig. 14). The expression of the five *AtVHA-c* subunits was unchanged except for *AtVHA-c3*, which was induced at 6 h (Fig. 15). At the same time, *ThVHAc1* showed much higher expression under the same conditions, especially at 12 h (Fig. 15), suggesting that expression of the exogenous *VHA-*c subunit may suppress the expression of intrinsic *VHA-c* genes. The expression patterns of all subunits were also similar in c1#17 (Fig. S4), indicating that expression of *ThVHAc1* may cause other subunits to participate in V-ATPase regulation under Cd stress and that V-ATPase activity is controlled by a complex network.

**Figure 16:**
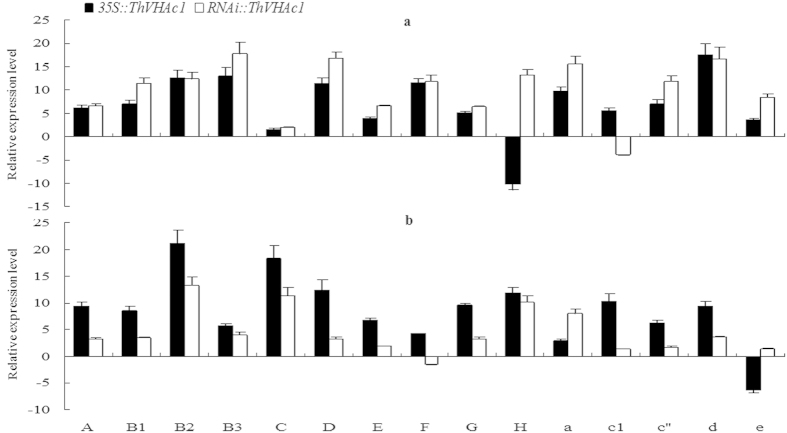
qRT-PCR analysis of *T. hispida* V-ATPase subunits in transient expression *ThVHAc1* lines. (**a**) Control conditions. (**b**) CdCl_2_ treatment. The relative expression levels were all log_2_ transformed. The X-axis shows the name of the V-ATPase subunit. The data are shown as the means ± SD of three independent experiments.

Interestingly, transient overexpression of *ThVHAc1* in *T. hispida* had a different effect. Fifteen subunits were amplified from the *T. hispida* cDNA library. All subunits except *ThVHA-H* in *35S::ThVHAc1* and *ThVHAc1* in *RNAi::ThVHAc1* showed positive expression levels under the control conditions, and most subunits showed greater expression in *RNAi::ThVHAc1* than in *35S*::*ThVHAc1* (Fig. 16). However, when treated with CdCl_2_, although all subunits except *ThVHA-e* in *35S::ThVHAc1* were induced, their expression was higher in *35S::ThVHAc1* than in *RNAi::ThVHAc1* (Fig. 16). These results suggest that *ThVHAc1* responds to Cd stress and that all subunits may participate in the regulation of V-ATPase activity. However, the mechanisms by which all subunits act in such a complex network of V-ATPase regulation require further study.

A previous study indicated that the expression patterns of different subunits of V-ATPase may differ under the same stress. The *Mesembryanthemum crystallinum* V-ATPase subunits A, B and c were all upregulated approximately 2-fold relative to the control plant in roots and young leaves when exposed to salt stress. However, when the leaves fully expanded, only the c subunit was induced in reaction to salt^66^. Sugar beet *VHA-A* and *VHA-c* were coordinately expressed during plant development and are induced in response to high salinity^67^. The subunit E was also induced after treatment with salt for 3 d in mature common ice plant leaves, but it was not induced in juvenile leaves under the same conditions^68^.

## Conclusion

In the plant kingdom, the V-ATPase c subunit (VHAc) is an important component of V-ATPase, which mediates abiotic stress responses. Some studies have demonstrated the salt regulation capacity of the *VHAc* gene. However, there are few reports on *VHAc* gene function in response to heavy metal stresses in a woody halophyte. Because *ThVHAc1* rapidly responded to Cd stress in *T. hispida*, in this study, we further investigated the role of *ThVHAc1* in Cd tolerance regulation. Our results showed that overexpression of *ThVHAc1* effectively enhanced the tolerance of the transgenic *Arabidopsis* and *T. hispida* plants to Cd stress and that *ThVHAc1* may modulate Cd stress tolerance by improving protective enzyme activities and strengthening the reactive oxygen species (ROS) scavenging system to decrease the cell damage when exposed to CdCl_2_ treatment. Moreover, we identified a potential upstream regulator of *ThVHAc1*, *ThWRKY7*, which also responded to Cd stress, showed expression patterns similar to those of *ThVHAc1*, and improved the Cd stress tolerance of transgenic *T.hispida*. Although it remains unclear whether *ThWRKY7* and *ThVHAc1* cooperate to participate in the regulation of tolerance to other stresses, the current study provides new insights into the role and regulatory mechanism of *ThVHAc1* in the regulation of tolerance to Cd stress in *T. hispida*.

## Additional Information

**How to cite this article**: Yang, G. *et al.* Overexpression of *ThVHAc1* and its potential upstream regulator, *ThWRKY7*, improved plant tolerance of Cadmium stress. *Sci. Rep.* **6**, 18752; doi: 10.1038/srep18752 (2016).

## Supplementary Material

Supplementary Information
