# Supplementary material for: Overexpression of ThVHAc1 and its potential upstream regulator, ThWRKY7, improved plant tolerance of Cadmium stress
Source: Sci Rep. 2016 Jan 8;6:18752. doi: 10.1038/srep18752 (PMC4705465; doi:10.1038/srep18752)

Title page

Overexpression of *ThVHAc1* and its potential upstream regulator, *ThWRKY7*, improved plant tolerance of Cd stress

Guiyan Yang[1,2], Chao Wang[1], Yucheng Wang[1], Yucong Guo[1], Yulin Zhao[1], Chuanping Yang[1]*, Caiqiu Gao[1]*

(1, State Key Laboratory of Tree Genetics and Breeding (Northeast Forestry University), 26 Hexing Road, Harbin 150040, China. 2, Laboratory of Walnut Research Center, College of Forestry, Northwest A & F University, Yangling, 712100 Shaanxi, China)

*Corresponding author: Chuanping Yang

E-mail: yangchuanpingnefu@yahoo.com

*Corresponding author: Caiqiu Gao

E-mail: gaocaiqiu@yahoo.com

Tel: +86-451-82191820

Fax: +86-451-82191822

Supplementary tables

Table S1 The primer sequences used in real-time RT-PCR and RT-PCR of *T.hispida*

Gene	Primer sequence	gene	Primer sequence
ThVHAc1	5’- GAGTGATGAGGCCAGAGTTG-3’<br>5’- ACCGGCATCACCAACAATACC -3’	ThVHA-B3	5’- ATGAGCGTGCTGGTCGTATTG -3’<br>5’- ATGGTCGCGACGAGTCATTC -3’
ThWRKY7	5’- CATAGGTCCAGACTCTTC -3’<br>5’- TTGTCCAGCTAGGCATCT -3’	ThVHA-B1	5’- GTACACAACCGTGCAGTTCAC -3’<br>5’- AGTTCATGACATCAATCGTAG -3’
ThSOD	5’- AGCTGTGGCAATTATCGC -3’<br>5’- CAATGATGTTACCCAGGT -3’	ThVHA-B2	5’- ATGGAATACAGGACTGTGTC -3’<br>5’- CCGAGCATGTCCTCTGACAC -3’
ThPOD	5’- CACCACCAGAAGAACAAATCG -3’<br>5’- CCCTAATGAAACAGTCATGG -3’	ThVHA-c”	5’- TGCCTCCATCGGTATAGCTATC -3’<br>5’- GATCTGAGTTGGTGATACACT -3’
ThGSTZ1	5’- GCCTGCACTTGGACTTAA -3’<br>5’- TCACGCTCTGTATCACCC -3’	ThVHA-C	5’- CTGCTTGCTCTCAGCGATGAT -3’<br>5’- CCTTCAGAGGAGACATGGTTG -3’
ThGPX	5’- CCAATTCTATCTACGATT -3’<br>5’- TCCTGGCTCTTGCCAAGC-3’	ThVHA-D	5’- CGCCTTAATGTTGTGCCTAC -3’<br>5’- CTCACCTGCAACATACTTGG -3’
Actin	5’- AAACAATGGCTGATGCTG -3’<br>5’- ACAATACCGTGCTCAATAGG -3’	ThVHA-d	5’- ATCTTCAGAATGAGCCTTCT -3’<br>5’- GCAGTGTTCCGGTTACAATC -3’
α-tubulin	5’- CACCCACCGTTGTTCCAG -3’<br>5’- ACCGTCGTCATCTTCACC -3’	ThVHA-E	5’- TCAATATCGAGAAGTTGCAG -3’<br>5’- CGATGATTGTCACCACTCAC -3’
β-tubulin	5’- GGAAGCCATAGAAAGACC -3’<br>5’- CAACAAATGTGGGATGCT -3’	ThVHA-e	5’- TCTAGTCGTTGGAATTATTGC -3’<br>5’-CTTCACTGTTCAGAATTGGCAC-3’
ThVHA-F	5’-CTGCAGACAGTTCAGAGTC-3’<br>5’-ACTTGCAACATTGTCACGC-3’	ThVHA-G	5’- TGTAATCTGGCTGTACAGCAT -3’<br>5’- TGGAACAGACCAGTTGATTCG -3’
ThVHA-A	5’- AGTTGCAGCGGAGTTACAAT -3’<br>5’- GATGCATCAGTAGACATACT-3’	ThVHA-H	5’- GTCAGCATTCTACGTGACAT -3’<br>5’- CCAGTATCTTACAGCTCCT -3’
ThVHA-a	5’-ATCTGGACATACTGCAATGC-3’<br>5’-TTCTGGAGCCACAGAACTCT-3’	CAX2	5’- GTATGATACGTGTTGTTC -3’<br>5’-AGTATGAGTCGAGTGAAGT-3’
NADPH	5’-CATTACACTTCGCAGTGT-3’<br>5’-AATCGACTACCGAGGACG-3’	CSB1	5’-ATGGCACTCGGTCTGTCC-3’<br>5’-ACCGAGTACGTTATACAT-3’
ADP	5’-TCATGTATTCGGCTATTG-3’<br>5’-TGGAGACGATGGTACCAC-3’	CSB2	5’-AGAGCATCTGCAGACCAG-3’<br>5’-AACAATTGCTATGGTTCC-3’
35S::ThVHAc1	5’-ATCGTCTAGAATGTCTACCGTATTCAACGGCG-3’<br>5’-CgATggTACCTTAGTCCGCTCTTGACTGTCCGGC-3’	CSB3	5’-CATGTGATAGGAGATTGT-3’<br>5’-GTGCAATCTTGCGCCTCT-3’
GLH	5’-CTGCTGCTCTTGATTGTGC-3’		5’-ATGGGCTGACCATATCCAT-3’

Table S2 The primer sequences used in one-hybrid assay and reporter and effector plasmids

**construct**	**Forward and Reverse Primers (5'-3')**	
pHis2-WRKY	AATTCGTGACAGTGACAGTGACAGAGCT	CTGTCACTGTCACTGTCACG
pHis2-WRKY-M	AATTCGGTCAAGGTCAAGGTCAAGAGCT	CTTGACCTTGACCTTGACCG
pHis2-WRKY-S	GGAATTCGTGACATTACACTTT	GGTCAATGTATACATAACGAGCTCG
pHis2-WRKY-S-M1	GGAATTCGATTACACTTTAAGAATGAAG	GATGTATACATAACCTTCTAGGGAGCTCG
pHis2-WRKY-S-M2	GGAATTCGGTCAATTACACTTTAAGAATG	GACTGATGTATACATAACCTTCTAGGGAGCTCG
pCAM-WRKY	AGCTTGTGACAGTGACAGTGACACCCTTC<br>CTCTATATAAGGAAGTTCATTTCATTTGGA<br>GAGAACACGGC	CATGGCCGTGTTCTCTCCAAATGAAATGAACTTCC<br>TTATATAGAGGAAGGGTGTCACTGTCACTGTCACA
pCAM-WRKY-M	AGCTTGGTCAAGGTCAAGGTCAACCCTTC<br>CTCTATATAAGGAAGTTCATTTCATTTGGA<br>GAGAACACGGC	CATGGCCGTGTTCTCTCCAAATGAAATGAACTTC<br>CTTATATAGAGGAAGGGTTGACCTTGACCTTGAC<br>CA
pCAM-WRKY-S	AGCTTGTGACATTACACTTT	CATGGCCGTGTTCTCTCCAAATGAAATGAACTTCC<br>TTATATAGAGGAAGGGCTATTTCTCATGTTTGAT
pCAM-WRKY-S-M1	AGCTTGATTACACTTTAAGAATGAAG	CATGGCCGTGTTCTCTCCAAATGAAATGAACTTCC<br>TTATATAGAGGAAGGGCTATTTCTCATGTTTGAT
pCAM-WRKY-S-M2	AGCTT GGTCAATTACACTTTAAGAATG	CATGGCCGTGTTCTCTCCAAATGAAATGAACTTCC<br>TTATATAGAGGAAGGGCTATTTCTCATGTTTGAT
prokII-ThWRKY7	ATCGTCTAGAATGGATGTCAGCGGTTTC	ATCGGAGCTC TCAAATGGGGAACTTTGGT

Table S3 The primer sequences used in real-time RT-PCR and RT-PCR of *Arabidopsis*

Gene	Primer sequence	gene	Primer sequence
At1g78900 (A)	5’- ATGGTATGGCCGGTGCTGCT-3’<br>5’- GGATATTCTTGCAATAGTCT -3’	At2g28520 (a3)	5’- CAGATATTGCACTTGGAG-3’<br>5’- ATCTCAATCCTGATTGAT-3’
At1g76030 (B1)	5’-TCTGCCAGAAGCATACCTTG-3’<br>5’-CCATGATCCTCAAGTAGATC-3’	At2g21410 (a1)	5’-GGAGTACAAGCTCGTTCTTG-3’<br>5’-CTGTCGTATGAAGATGTTGC-3’
At4g38510 (B2)	5’- TCTGGAAGTTCTATCAATC-3’<br>5’- CATTGCTGCGAACACAATG-3’	At4g39080 (a2)	5’-TGACATTGACTTGGATGATG-3’<br>5’-GTCGAATCAATGGACTTCTC-3’
At1g20260 (B3)	5’-CTGTGTCATTGGACATGCTT-3’<br>5’-CCACGAGCAATGGAGTTCAT-3’	At4g34720 (c1)	5’-GGAGTTATGAGACCTGAGT-3’<br>5’-AATGGCCATTCCAGCTGAG-3’
At1g12840 (C)	5’-CGATGATCTGCTTAAGTCG-3’<br>5’-GAACCTTGAGATCATCCTC-3’	At1g19910 (c2)	5’-GTGTGATGAGACCAGAGCT-3’<br>5’-GTTGTGCATTCGCTCTAAC-3’
At3g58730 (D)	5’-TACAGCTAAGGAGTCTATG-3’<br>5’-CTGTTGACCACCTCTAGCT-3’	At4g38920 (c3)	5’-CTCATGTATGGGAGCTGCGT-3’<br>5’-CATCGCCAACGATACCTATG-3’
At4g11150 (E1)	5’-GATTGATTACTCTATGCAGC-3’<br>5’-CATCAAGTACAGCTTCCACT-3’	At1g75630 (c4)	5’-GAGTGATGAGACCTGAACT-3’<br>5’-AATCAGGATTACAATCAAC-3’
At3g08560 (E2)	5’- CTCGAGTCTGCCAAGCGT -3’<br>5’- TCTCAATGATGAGACTCT -3’	At4g26710 (e2)	5’-ATGAGAGGGTTCATCTGCG-3’<br>5’-CGACGATCCTCCATCATCC-3’
At1g64200 (E3)	5’-AGATTGATTACTCTATGCAG-3’<br>5’-CATCAAGTACAGCTTCCACT-3’	At2g16510 (c5)	5’-GGAGTGATGAGACCTGAGT-3’<br>5’-ATACCAGCTGAGAGTCCAG-3’
At3g42050 (H)	5’- GTTAGCATCTTACGTGATAT-3’<br>5’- CCAATAACAGCATTACCAGC-3’	At4g32530 (c"1)	5’-AATCTCATCAGTGTCATCT-3’<br>5’-AACAACCCGAGAGCACTCC-3’
At4g25950 (G3)	5’-GACGGATTGTTTCAGCTGCG-3’<br>5’-TACTTGATGAGCATCTTGAC-3’	At2g25610 (c"2)	5’-GAATCTCATCAGTGTAATCT-3’<br>5’-AGCGTCGAGTTCTGAGCATC-3’
At3g01390 (G1)	5’-TGAGGTAGAAGCTCAACAC-3’<br>5’-CTTCGTTCTTCAACTGCTCG-3’	At3g28715 (d2)	5’-ATCTATGTCAGTGTGAGAAC-3’<br>5’-GACCATATCTGATGTACTCT-3’
At4g02620 (F)	5’-TCATCGCAATGATCGCCGAT-3’<br>5’-ATCTCCAAGATTGCAGGAAC-3’	At5g55290 (e1)	5’-ATAACAACCCTAATCTTCG-3’<br>5’-CTACTCTGTTTCGCTTAGA-3’
At4g23710 (G2)	5’-ATCCAGCAACTGCTTGCTGC-3’<br>5’-CAGTTGTTCACTGTGGTCAC-3’	At3g28710 (d1)	5’-ATCGTTAGAGGCCACCGTG-3’<br>5’-AGACATTGGCTCAGTAGCT-3’
At2g07560 (AHA)	5’-GTCTTCAGAGGAAGGAAG-3’<br>5’-GTCGAGTTAATGATGAGC-3’	At3g18780	5’-TGCAGGAGATGATGCTCC-3’<br>5’-ATACGAAGCTCATTGTAG-3’
At1g80660 (AHA)	5’-TATCATCAACTCTACCAT-3’<br>5’-TCTTGAGAGGATCACCAT-3’	At1g08830 (*CSD*)	5’-ATGGTGTGACCACTGTGAG-3’<br>5’-GAATCTGGCAATCAGTGAT-3’
At3g42640 (AHA)	5’-GACTAAGGTGTTGAGAGAT-3’<br>5’-CTTGCTTGCAGGTTGAGC-3’	At1g07890 (*APX*)	5’-CAGGAGGAAGCTCAGAGG-3’<br>5’-AACACCAGCAAGCTGATG-3’
At1g27770 (ACA)	5’-AGTGAGTATACACTACCT-3’<br>5’-CTGTGAATTGGTTGATTC-3’	At3g45140 (*LOX1*)	5’-GATTCAGAACCAATACCAT-3’<br>5’-TAAGGTCTCGAGCTCCTC-3’
At1g08065 (ACA)	5’-ACATGCAGTCTCCGATTG-3’<br>5’-TTAATATAGTTTACTATGC-3’	At5g51060 (*RBOHC*)	5’-ACTGCTGAGAACAGTCAG-3’<br>5’-AACGCTTAAGCTCGTGAG-3’
At1g08080 (ACA)	5’-ATGAACGAGAGAGTTAAC-3’<br>5’-ATTAATAGTATGTTCAGAC-3’		

Supplementary Fig.S1 Schematic map of the recombsinant vectors. a, Schematic map of the ThVHAc1 promoter inserted in the pCAMBIA1301 binary vector, which was used for Arabidopsis transformation. b, Diagram of ThVHAc1 promoter showing the WRKY motif. c, Structure of reporter and effector vector. Cis×3, three WRKY motifs. m-Cis×3, three mutant WRKY motifs. △promoter, a fragment of promoter containing the WRKY motif. △m-promoter, a fragment of promoter containing the mutated WRKY motif or excluding the motif. d, Diagram of the reporter and effector constructs used in the transient co-expression in Arabidopsis.

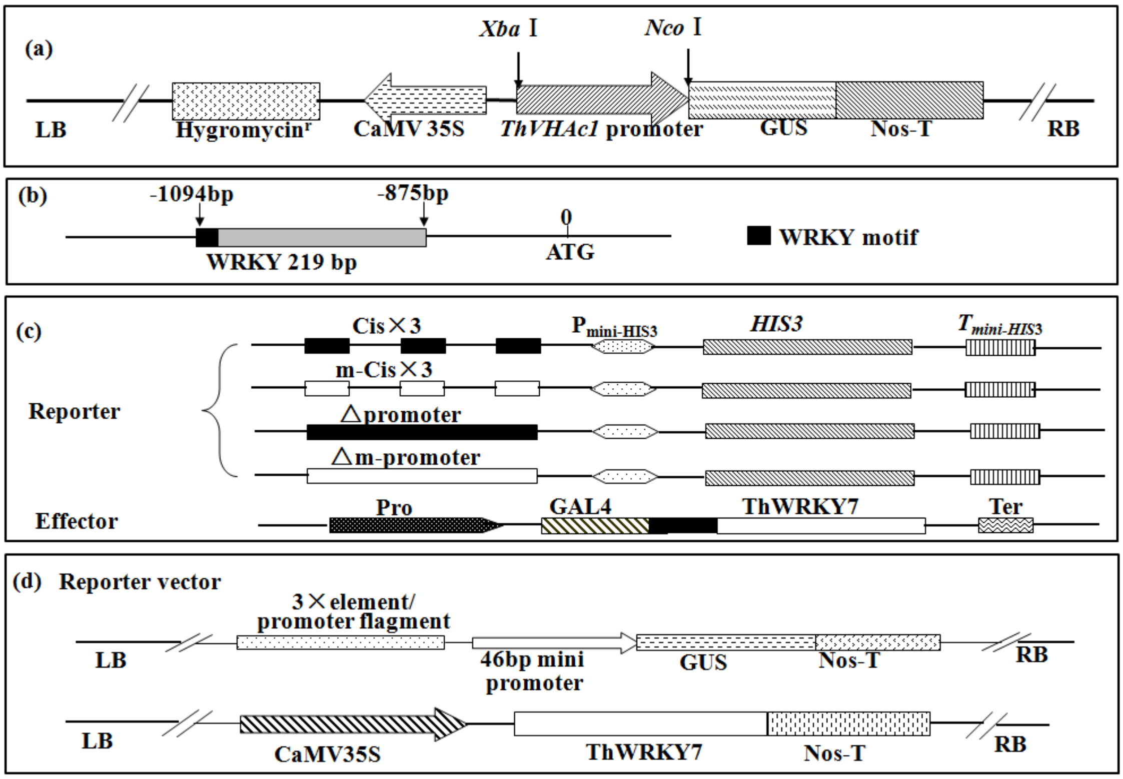

Supplementary Fig.S2 Mapping of the cis-elements in the *ThVHAc1* promoter

ATCGAGTATGGTGTTATGGAAGCGCATAAGAAGCAGGTATGAGAGGGTGACAATCTA
CACTFTPPCA1 WRKY710S CAATBOX1
TAGCAGCTCGGTGGTGACATTACACTTTAAGAATGAAGAACACCCCATCTCAGTAG
ANAERO2CONSENSUS GTGABOX
ACATACCTCACTCTTGGATGCATGCAAGCATTAGATGAGTTAGTTTTATTTGTATTAG
NODCON2GM CACTFTPPCA1 CACTFTPPCA1 TATABOX5
GTGGTTCTTAACTGGACAAACCATGCACCAGATGCTTTAGTCCACTTGGAAGTTGG
MYB1AT
AAGTCAGAGTAGAAGCTTGAAATCCATGCATATGTGAACCTCCTAGAAGGTTATGTA
CACTFTPPCA1 CATATGGMSAUK
TACATTGACCTTTTGATCTAGTAGGTGGATGTGCATTAGGTCTATAGTTAACCAACAT
WRKY710S CACTFTPPCA1 MYB1AT
GGGTGTCTTTTTTTTTTTTTTATATTGTAAATACTGGATTGTATCCTCCTCAATCCATG
ARR1AT CAATBOX1
AACAATTAAAATAAAAAGGAGCCTTTTGACTAAAAAAAGAAATAAACATGAAATTC
CAATBOX1 POLASTG1 DOFCOREZM WRKY710S DOFCOREZM POLASTG1
ACACTACTAAAATATTTCCTCGATGTCCAAATAACATCCTATTTGATATGAAATCGAG
LECPCEACS2 GATABOX
TAGGAATTGGAAATCAATTTCTAATTCCTTAAATTTAGGATTCTATTTTTATTGCCTAG
CAATBOX1 ARR1AT
AATTTAATGGAAGATTTGTATAACTCAATAAAATATTTATTTTTATATATCAAACATGA
CAATBOX1 LECPCEACS2 TATABOX5
GAAATAGAATTTTATTTTTATAATTTTTATCCATCTCAAATAAAGCGAAAATGAAGGA
BOXIINTPATPB TATABOX5 AMYBOX2 POLASTG1 DOFCOREZM
GGTAAGCAGATAAATAGAAACTTAAGACTTGGACTTGGTTCTAGTTGGATCATTCCA
GATABOX BOXIINTPATPB
ACAAAAATGGACAAATACAGTTGAGTATCGGAAAAACGCCCCAACTTGAGACCATG

TTATTCACCTAAACAGCCCAAATCAATAGGCCGAAACTTAGATCTTTACTACGATCTT
CAATBOX1
TCCTCACACCAGTCCAAAGCAAATAATTACTAAAAAACAAACAAGGAAAAAATAAA
DOFCOREZM AACACOREOSGLUB1 ANAERO1CONSENSUS POLASTG1
ATAAAACAAATCAATAAAGTAAAATATGCTAAAATCTACGATCGTCAGATCACCATA
ANAERO1CONSENSUS CAATBOX1 DOFCOREZM LECPCEACS2
CAAAAAACAAATGAATGGGATTGTCCTCTACTTCATATGCATCACGCTGCCCAGATA
ANAERO1CONSENSUS ARR1AT CATATGGMSAUK CACTFTPPCA1 GATABOX
ACTAAAACCCACGACATCTCTGCGACCCCCCTCTCTTTTTCTCGCCCTAATTCTTCAT
NODCON2GM
AAATCTCTCAGATCCATCTCCAGTCAATCTCAATTCTAATCAAAAATAAGTTCTTCCA
CAATBOX1 CAATBOX1
CCTTATTTCTCCTATA
TATABOX5

Supplementary Fig.S3 Transient expression analyzed of ThWRKY7 in *T. hispida* under 100 μM $CdCl_2$ for 1 h and 2 h. a, DAB staining. b, Evans blue staining. c, qRT-PCR analysis of *ThWRKY7* in transient 35S::ThWRKY7 *T. hispida* seedlings based on T-ck. The related expression levels were all log2 transformed. d-i, the SOD, POD, GST, GPX activities, MDA content and EL of T-ck and 35S::ThWRKY7 seedlings. All experiments were repeated three times. The data are the means ±SD of three independent experiments.

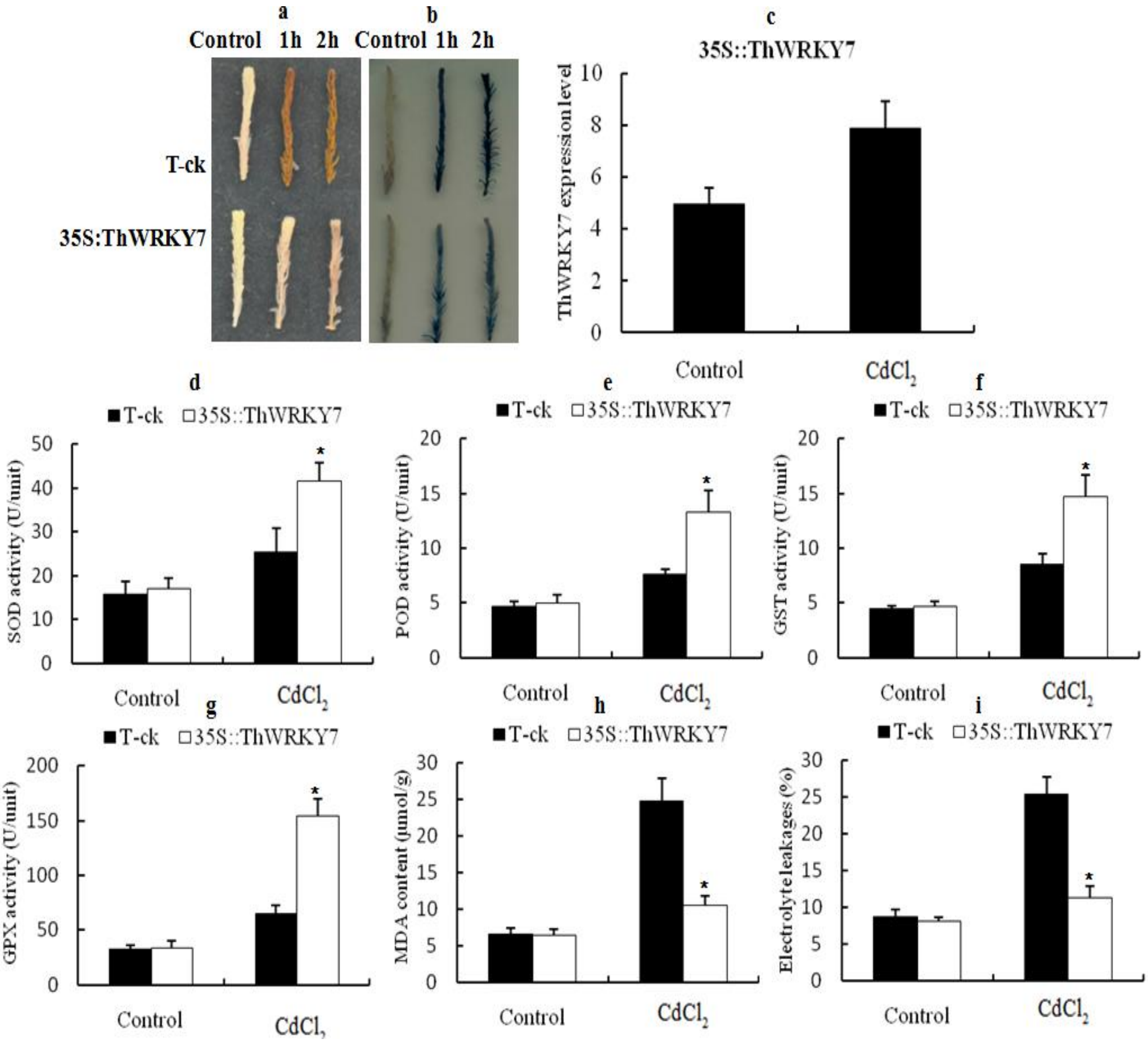

46 Supplementary Fig.S4 Cluster analysis of c1#17

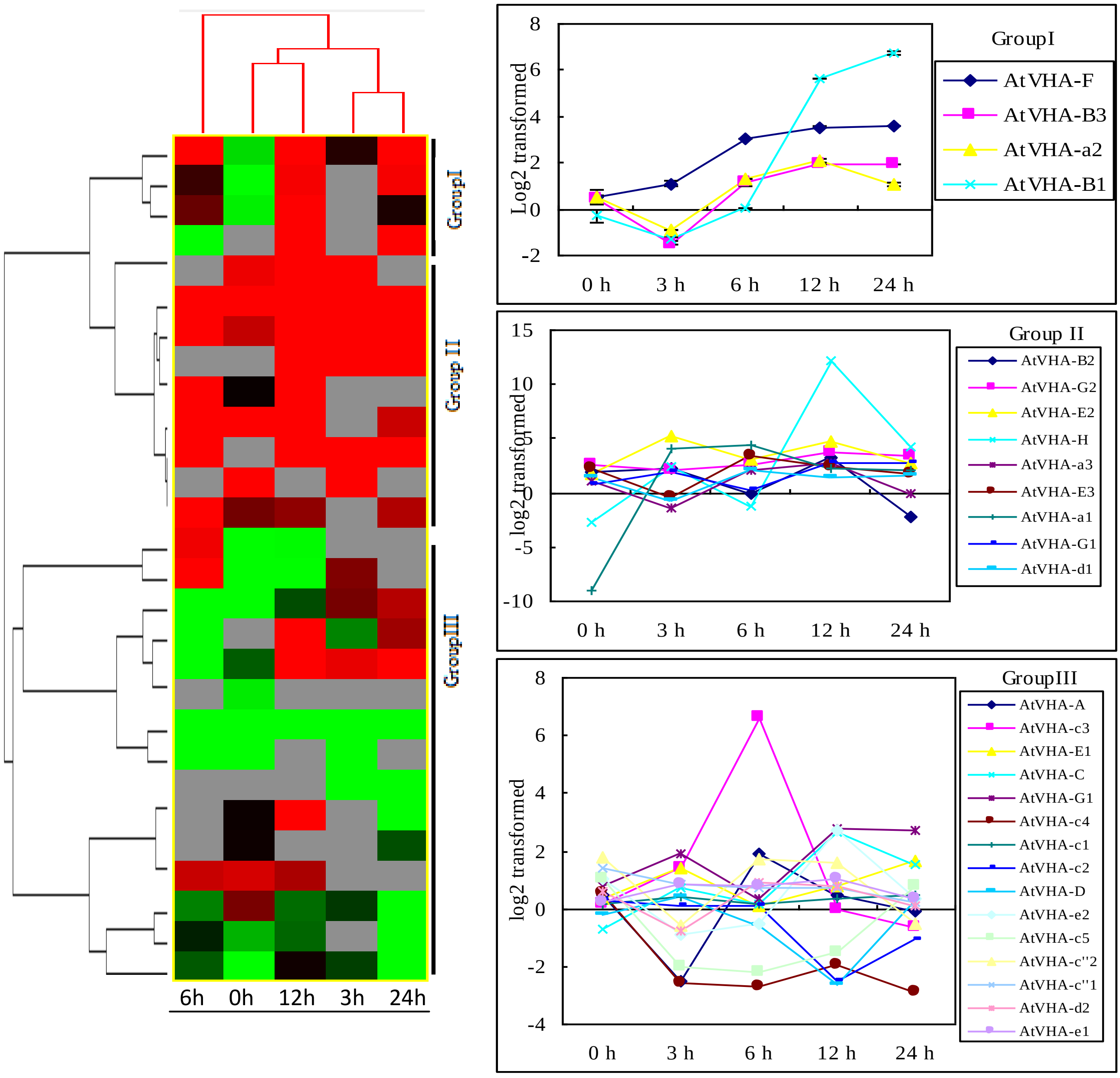

Supplement: Supplementary Information [file srep18752-s1.pdf]
